# Functional Analysis of Cancer-Associated DNA Polymerase ε Variants in *Saccharomyces cerevisiae*

**DOI:** 10.1534/g3.118.200042

**Published:** 2018-01-19

**Authors:** Stephanie R. Barbari, Daniel P. Kane, Elizabeth A. Moore, Polina V. Shcherbakova

**Affiliations:** Eppley Institute for Research in Cancer and Allied Diseases, Fred & Pamela Buffett Cancer Center, University of Nebraska Medical Center, Omaha, Nebraska 68198

**Keywords:** DNA polymerase ε, *POLE*, cancer, mutator, proofreading

## Abstract

DNA replication fidelity relies on base selectivity of the replicative DNA polymerases, exonucleolytic proofreading, and postreplicative DNA mismatch repair (MMR). Ultramutated human cancers without MMR defects carry alterations in the exonuclease domain of DNA polymerase ε (Polε). They have been hypothesized to result from defective proofreading. However, modeling of the most common variant, Polε-P286R, in yeast produced an unexpectedly strong mutator effect that exceeded the effect of proofreading deficiency by two orders of magnitude and indicated the involvement of other infidelity factors. The *in vivo* consequences of many additional Polε mutations reported in cancers remain poorly understood. Here, we genetically characterized 13 cancer-associated Polε variants in the yeast system. Only variants directly altering the DNA binding cleft in the exonuclease domain elevated the mutation rate. Among these, frequently recurring variants were stronger mutators than rare variants, in agreement with the idea that mutator phenotype has a causative role in tumorigenesis. In nearly all cases, the mutator effects exceeded those of an exonuclease-null allele, suggesting that mechanisms distinct from loss of proofreading may drive the genome instability in most ultramutated tumors. All mutator alleles were semidominant, supporting the view that heterozygosity for the polymerase mutations is sufficient for tumor development. In contrast to the DNA binding cleft alterations, peripherally located variants, including a highly recurrent V411L, did not significantly elevate mutagenesis. Finally, the analysis of Polε variants found in MMR-deficient tumors suggested that the majority cause no mutator phenotype alone but some can synergize with MMR deficiency to increase the mutation rate.

In normal cells, DNA is replicated with great accuracy to avoid mutations that can lead to disease. High-fidelity DNA replication is achieved through the correct nucleotide selection by replicative DNA polymerases α (Polα), δ (Polδ), and ε (Polε), exonucleolytic proofreading by Polδ and Polε, and postreplicative DNA mismatch repair (MMR) (Ganai and Johansson 2016). It has long been known that germline mutations in MMR genes cause predisposition to colorectal cancer (CRC) and an increased risk of several other cancer types in Lynch syndrome (Peltomäki 2003). Somatic MMR defects are also common in sporadic CRC, endometrial cancer (EC), and gastric cancer. By contrast, although impaired nucleotide selectivity and proofreading promote tumorigenesis in mouse models (Goldsby *et al.* 2001; Venkatesan *et al.* 2007; Albertson *et al.* 2009), the association between replicative DNA polymerase defects and cancer in humans has not been established until recently. Several years ago, large-scale molecular characterization of sporadic CRC and EC by the Cancer Genome Atlas Network identified a subset of tumors that were significantly hypermutated (>10 mutations per megabase). Many of them, as expected, displayed microsatellite instability (MSI) indicative of MMR defects. However, the most hypermutated tumors were microsatellite stable (MSS) and contained somatic mutations in the *POLE* gene encoding the catalytic subunit of Polε (Cancer Genome Atlas Network 2012; Cancer Genome Atlas Research Network 2013). Many subsequent whole-exome and targeted sequencing studies of CRC and EC samples similarly reported somatic *POLE* mutations, together revealing that at least 6% of colorectal and 7% of endometrial tumors contain these changes (Barbari and Shcherbakova 2017). *POLE* mutations have also been observed, although less frequently, in other types of gastrointestinal cancer, as well as tumors of the brain, breast, ovary, prostate, lung, kidney, cervix, and bone (Cerami *et al.* 2012; Forbes *et al.* 2015; Grossman *et al.* 2016; Campbell *et al.* 2017). In addition to somatic defects, germline *POLE* mutations were found in patients with hereditary CRC, with strong evidence for the causative role of a highly penetrant *POLE-L424V* variant predisposing to multiple colorectal adenomas and carcinomas (Palles *et al.* 2013).

*POLE* is an essential gene, and nearly all variants reported in cancers are missense mutations, for which the pathogenicity cannot be definitively predicted in the absence of functional analyses. The amino acid substitutions, however, tend to cluster in the exonuclease domain, with recurring hotspots at highly conserved residues. Many of these substitutions were predicted by *in silico* analysis to affect DNA binding and/or exonuclease activity (Church *et al.* 2013; Palles *et al.* 2013; Rohlin *et al.* 2014; Hansen *et al.* 2015; Rayner *et al.* 2016). These findings have led to the notion that faulty proofreading is the main consequence of the polymerase alterations and is therefore responsible for the hypermutation observed in the tumors. Indeed, *in vitro* assays have shown that several cancer-associated Polε variants have reduced exonuclease activity and fidelity (Shinbrot *et al.* 2014). Additionally, the presence of these variants in tumors is significantly correlated with a hypermutated phenotype and a unique mutation signature, consistent with a primary role of the polymerase defects in driving the hypermutation (Alexandrov *et al.* 2013; Church *et al.* 2013; Shinbrot *et al.* 2014; Shlien *et al.* 2015; Campbell *et al.* 2017). Yet, several observations are difficult to reconcile with the idea that defective proofreading is the sole consequence of Polε mutations. First, some variants are seen at a vastly greater frequency than others, despite similar effects on exonuclease activity. Second, changes of catalytic residues in the exonuclease domain are rarely reported. Functional studies *in vivo* have only been performed for the most frequently recurring Polε-P286R substitution (Kane and Shcherbakova 2014). When modeled in yeast, this variant has an extraordinary mutator effect that exceeds the effect of proofreading deficiency by two orders of magnitude, supporting a pathogenic role but also suggesting a consequence beyond defective proofreading alone. This striking phenotype indicates that further mechanistic and functional *in vivo* studies are required to fully understand the impact of cancer-associated Polε mutations.

Although Polε-P286R is the most common variant in cancers, additional, less frequent variants likely account for a large proportion of disease cases. In this work, we assess the functional consequences of 13 additional Polε variants in the genetically tractable yeast system. We show that only mutations affecting residues near the DNA binding cleft in the exonuclease domain have mutator effects. The magnitude of the mutator effects is highly variable, but in the vast majority of cases it exceeds the effect of proofreading deficiency. Furthermore, frequently recurring DNA binding cleft variants had stronger mutator effects than rarer variants, in line with the idea that the likelihood of developing cancer is proportional to the severity of the mutator phenotype. This finding suggests that the vastly different frequencies of Polε variants in sporadic tumors can be explained, at least in part, by differences in their mutator effects. We further validate the pathogenicity of DNA binding cleft mutations by demonstrating their ability to confer a mutator phenotype in the heterozygous state, which mimics the state of Polε alterations in tumors. Finally, analysis of several Polε variants found in MMR^−^ tumors suggests that although many such variants are likely to be neutral passenger mutations, some could be weak mutators that synergize with MMR deficiency to promote genome instability.

## Materials and Methods

### Yeast strains and plasmids

All *Saccharomyces cerevisiae* strains (Supplemental Material, Table S1 in File S1) were derived from TM30 (*MAT*a *ade5-1 lys2-Tn5-13 trp1-289 his7-2 leu2-3*,*112 ura3-4 CAN1*::*LEU2*) and/or TM44 (*MATα ade5-1 lys2*::*InsE_A14_trp1-289 his7-2 leu2-3*,*112 ura3-52 can1*Δ::*loxP*) (Mertz *et al.* 2015). Mutations in the *POL2* gene encoding the catalytic subunit of Polε were created by site-directed mutagenesis in one of the following *URA3*-based integrative vectors. YIpDK1 contains the 2.1-kb N-terminal *Hpa*I-*Eco*RI fragment of *POL2*, which includes the promoter and exonuclease domain regions (Kane and Shcherbakova 2014). p173 contains the 5.5-kb C-terminal *Bam*HI-*Bsp*EI fragment of *POL2* including the DNA polymerase domain and downstream flanking DNA (Pavlov *et al.* 2001). p174 contains the 9.8-kb C-terminal *Bam*H1-*Sst*I fragment of *POL2* including the DNA polymerase domain and a more extended downstream flanking region (Pavlov *et al.* 2001). YIpDK1 was used to make *pol2-F139L*, *pol2-R252H*, *pol2-D290V*, *pol2-P301H*, *pol2-N351S*, *pol2-F382S*, *pol2-V426L*, *pol2-L439V*, *pol2-P451R*, and *pol2-S474F*; p173 was used to make *pol2-R778W* and *pol2-A979V*; and p174 was used to make *pol2-D1757N*.

Haploid strains were constructed by one of two methods, both of which consider the possibility that the variant alleles might result in lethality if expressed as the sole source of the polymerase. In both methods, the mutant alleles are first introduced into cells that also express the wild-type *POL2*, and the viability of the mutants is assessed after a loss of the wild-type allele is allowed to occur in nonselective conditions. Both methods ultimately result in variant alleles expressed at their natural chromosomal locations from the endogenous promoter. In the first method, TM63 diploid (created by crossing TM30 with TM44) was transformed with an integrative plasmid (YIpDK1-pol2-x, p173-pol2-x, or p174-pol2-x) linearized with the appropriate restriction enzyme (Table S2 in File S1) such that integration into one of the two *POL2* loci placed the *URA3* selection marker between a full-length copy of *POL2* with the mutation and a truncated copy of *POL2* without the mutation. These diploids were then sporulated, and haploids were generated by tetrad dissection. The presence of four viable haploid spores in each tetrad indicated that the *pol2* mutation was not lethal. Derivatives of the Ura^+^ (mutant) haploids that underwent recombination to lose the *URA3* marker and restore a single, full-length *POL2* or *pol2-x* allele were then selected for on media containing 5-fluoroorotic acid. Clones with the *pol2-x* mutant allele were identified by sequencing. Diploid strains heterozygous or homozygous for the *pol2-x* mutation were created by crossing *CAN1*::*LEU2 pol2-x* haploids with *can1*Δ *POL2* or *can1*Δ *pol2-x* haploids of the opposite mating type, respectively. The diploids contain the *Kluyveromyces lactis LEU2* gene downstream of the *CAN1* gene in one chromosome and a deletion of *CAN1* in the homologous chromosome, allowing for the selection of recessive *can1* mutants on medium containing canavanine and lacking leucine (Mertz *et al.* 2015).

In the second method, haploids (TM30 and TM44) were transformed with an integrative plasmid linearized with the appropriate restriction enzyme (Table S2 in File S1) such that integration would produce strains containing the *URA3* selection marker between a full-length copy of *POL2* without the mutation and a truncated copy of *POL2* with the mutation. These strains were further transformed with a YEpPOL2-trp plasmid constructed by cloning the 12.1-kb *Xho*I-*Sst*I fragment from YEpPOL2 (Araki *et al.* 1992) containing the entire open reading frame of *POL2* plus flanking DNA into the *Sal*I and *Sst*I sites of YEplac112 (Gietz and Sugino 1988). The YEpPOL2-trp plasmid provided ectopic expression of wild-type *POL2*. Clones that underwent recombination at the chromosomal *POL2* locus to lose the *URA3* marker and restore a single full-length *POL2* or *pol2-x* allele were then selected for on media containing 5-fluoroorotic acid. Recombinants with the *pol2-x* mutant allele were identified by sequencing. Their ability to lose the YEpPOL2-trp plasmid on nonselective media was determined as previously described (Daee *et al.* 2010). A high frequency of plasmid loss indicated that the *pol2-x* mutation was not lethal. The Trp^−^ derivatives containing only the chromosomal mutant allele were used for the mutation rate measurements. Diploids heterozygous or homozygous for the *pol2-x* mutations were created by crossing TM30 *pol2-x* with TM44 *POL2* or TM44 *pol2-x*, respectively.

To generate strains lacking *MLH1*, wild-type and *pol2-x* mutant haploid strains were transformed with a PCR-generated *mlh1*Δ::*hphMX4* deletion cassette as previously described (Goldstein and McCusker 1999). Diploid strains homozygous for the *MLH1* deletion were generated by crossing *CAN1*::*LEU2pol2-x mlh1*Δ::*hphMX4* strains with *can1*Δ *POL2mlh1*Δ::*hphMX4* strains of the opposite mating type.

### Spontaneous mutation rate measurements

The rate of spontaneous mutation to canavanine resistance (Can^R^) was measured by fluctuation analysis as described (Northam *et al.* 2010). The rates of *his7-2* and *lys2*::*InsE_A14_* reversion were determined similarly, except that the revertants were selected on synthetic complete medium lacking histidine or lysine, respectively. To measure the rate of Can^R^ mutation in diploids, mutants were selected on synthetic complete medium lacking arginine and leucine, and containing 60 mg/liter L-canavanine. Mutation rates are reported as the median for at least 18 independent cultures of each strain. The 95% confidence interval for the median was determined as previously described (Dixon and Massey 1969; Van der Parren 1970). The significance of differences between mutation rates was determined by the Wilcoxon–Mann–Whitney nonparametric test.

### Frequency of POLE variants in tumors

The *POLE* variant frequency was calculated from published studies of sporadic CRC and EC (a total of >13,000 tumors; Table S4 in File S1). The number of documented occurrences of each variant was divided by the total number of tumors in which the corresponding exon of *POLE* was sequenced. Data on CRC and EC were combined. *P* values for pairwise comparison of frequencies of individual variants were calculated by Fisher’s exact test.

### Data availability

Strains and plasmids are available upon request. All mutation rate and variant frequency data used to reach the conclusions are presented fully within the article and the supplemental material.

## Results

### Cancer-associated Polε mutations have varying mutator effects

**Figure 1:**
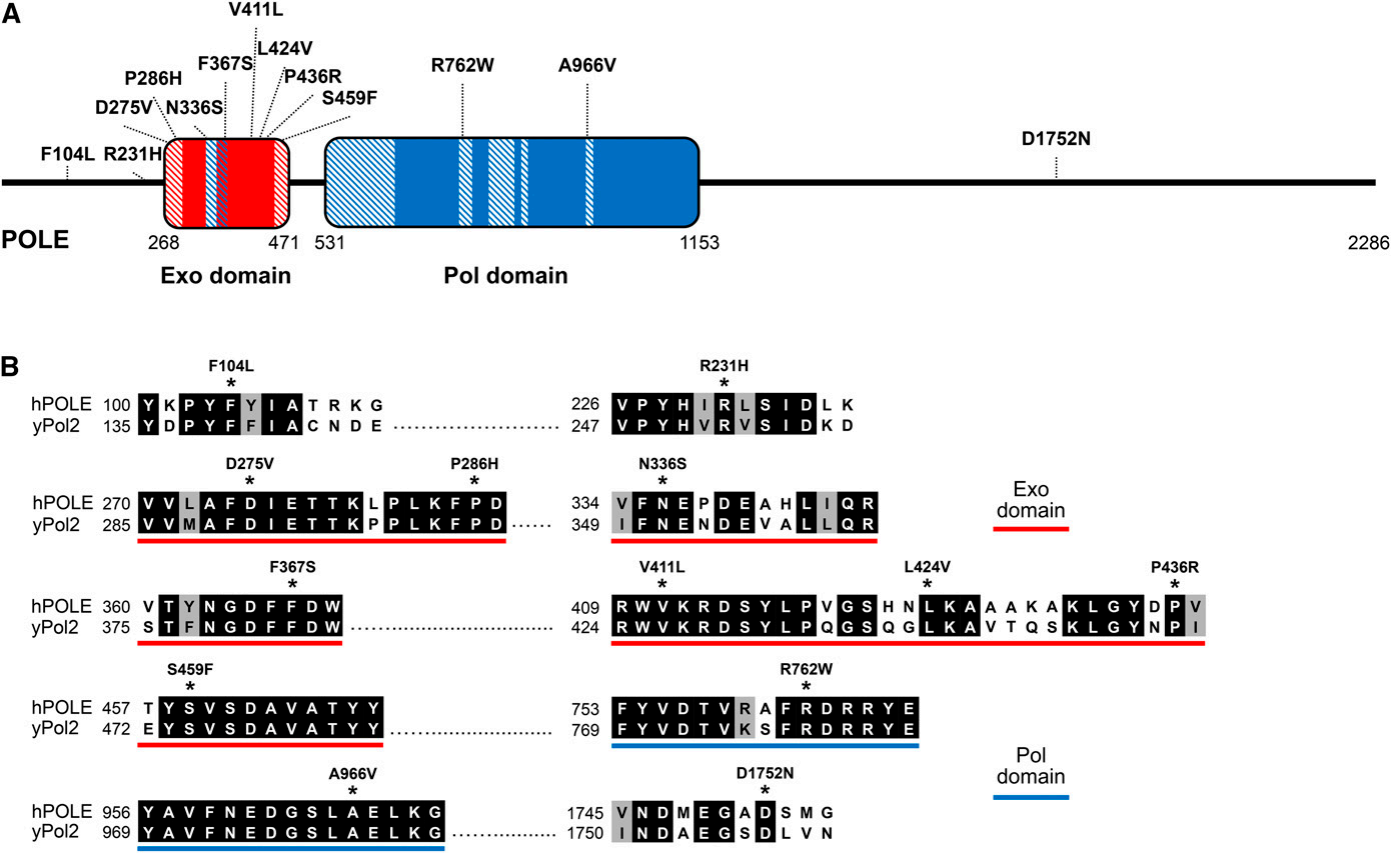
Cancer-associated Polε mutations studied in this work. (A) Schematic of human POLE showing the exonuclease (Exo) and polymerase (Pol) domains, conserved Exo and Pol motifs (hatched boxes), and the locations of mutations. (B) Alignment of amino acid sequences of human POLE and yeast Pol2 around the mutation sites.

The DNA replication machinery is structurally and functionally conserved among eukaryotes, and the yeast and human Polε show a high degree of amino acid sequence similarity. Thus, *in vivo* consequences of human Polε variants can be evaluated by studying analogous mutations in yeast, which is easily amenable to genetic manipulation and offers a variety of well-controlled mutagenesis assays. Several hundred distinct Polε mutations have been reported in human tumors (Barbari and Shcherbakova 2017; Campbell *et al.* 2017). The set of 13 Polε variants studied in this work (Figure 1 and Table 1) included seven variants within the exonuclease domain (D275V, P286H, F367S, V411L, L424V, P436R, and S459F); five variants outside the exonuclease domain that were found in hypermutated MMR-deficient (MSI) tumors (R231H, R762W, and A966V) or hypermutated MMR-proficient (MSS) tumors (F104L and D1752N); and a single nucleotide polymorphism (SNP) in the exonuclease domain, N336S, which is present in the general population at a frequency of 0.9%. Most exonuclease domain variants have been suggested to be pathogenic based on their recurrence in hypermutated tumors, predicted effects on DNA binding or catalysis, and/or demonstrated exonuclease defects *in vitro* (Church *et al.* 2013; Shinbrot *et al.* 2014; Rayner *et al.* 2016; Campbell *et al.* 2017).

**Table 1:** Characteristics of Polε mutations studied in this work

Classification	Human POLE Variant	Tumor Types	Mutation Origin	No. Times Reported*^a^*	MMR Status of Tumors	References
Exo domain, at DNA binding cleft	D275V	EC, GBM, LuC	Somatic	3	MSS	Church *et al.* (2013), Campbell *et al.* (2017)
	P286H	CRC, GBM	Somatic	2	MSS	Cancer Genome Atlas Network (2012), Campbell *et al.* (2017)
	F367S	CRC, EC	Somatic	5	MSS&MSI	Yoshida *et al.* (2011), Cancer Genome Atlas Network (2012), Grossman *et al.* (2016), McConechy *et al.* (2016)
	L424V	CRC, EC, BrC, GBM	Somatic	5	MSS	Cancer Genome Atlas Research Network (2013), Shinbrot *et al.* (2014), Stenzinger *et al.* (2014), Köbel *et al.* (2016), Andrianova *et al.* (2017)
		CRC, EC, LC, DuC, OC, GBM	Germline	24	MSS	Palles *et al.* (2013), Valle *et al.* (2014), Chubb *et al.* (2015), Elsayed *et al.* (2015), Spier *et al.* (2015), Johanns *et al.* (2016)
	P436R	EC, CRC	Somatic	7	MSS	Cancer Genome Atlas Network (2012), Billingsley *et al.* (2015), Talhouk *et al.* (2015), Grossman *et al.* (2016), McConechy *et al.* (2016), Campbell *et al.* (2017)
	S459F	CRC, EC, HGG, AA, GBM, DuC	Somatic	27	MSS	Cancer Genome Atlas Network (2012), Stenzinger *et al.* (2014), Erson-Omay *et al.* (2015), Shlien *et al.* (2015), Domingo *et al.* (2016), Giannakis *et al.* (2016), Grossman *et al.* (2016), Jansen *et al.* (2016), Wong *et al.* (2016), Campbell *et al.* (2017), Zehir *et al.* (2017)
Exo domain, distant from DNA binding cleft	V411L	EC, CRC, USC, GBM, OC, STAD, HGG, KC, PC	Somatic	122	MSS	Cancer Genome Atlas Network (2012), Church *et al.* (2013, 2015), Cancer Genome Atlas Research Network (2013), Zhao *et al.* (2013), Meng *et al.* (2014), Shinbrot *et al.* (2014), Billingsley *et al.* (2015), Erson-Omay *et al.* (2015), Hoang *et al.* (2015), Talhouk *et al.* (2015), Domingo *et al.* (2016), Giannakis *et al.* (2016), Jansen *et al.* (2016), McConechy *et al.* (2016), Mehnert *et al.* (2016), Wong *et al.* (2016), Campbell *et al.* (2017), Espinosa *et al.* (2017), Gong *et al.* (2017), Zehir *et al.* (2017)
		CRC	Germline	1	MSS	Wimmer *et al.* (2017)
Outside Exo domain, MSI tumors	R231H	CRC	Somatic	1	MSI	Cancer Genome Atlas Network (2012)
	R762W	CRC	Somatic	1	MSI	Cancer Genome Atlas Network (2012)
	A966V	CRC	Somatic	1	MSI	Seshagiri *et al.* (2012)
Outside Exo domain, MSS tumors	F104L	CRC, PXA	Somatic	2	MSS	Seshagiri *et al.* (2012), Shlien *et al.* (2015)
	D1752N	CRC	Somatic	1	MSS	Cancer Genome Atlas Network (2012)
SNP	N336S	—	Germline	—	—	NCBI SNP database

a Indicates the number of times the variant has been reported in whole-exome or targeted sequencing studies of all cancer types. Because many studies analyzed only selected exons of *POLE*, these numbers do not necessarily reflect the relative frequency of each variant.

AA, anaplastic astrocytoma; BrC, breast carcinoma; CRC, colorectal cancer; DuC, duodenal carcinoma; EC, endometrial cancer; GBM, glioblastoma; HGG, high grade glioma; KC, kidney cancer; LC, liver cancer; LuC, lung cancer; OC, ovarian cancer; PC, prostate cancer; PXA, pleomorphic xanthoastrocytoma; STAD, stomach adenocarcinoma; USC, uterine serous carcinoma.

**Figure 2:**
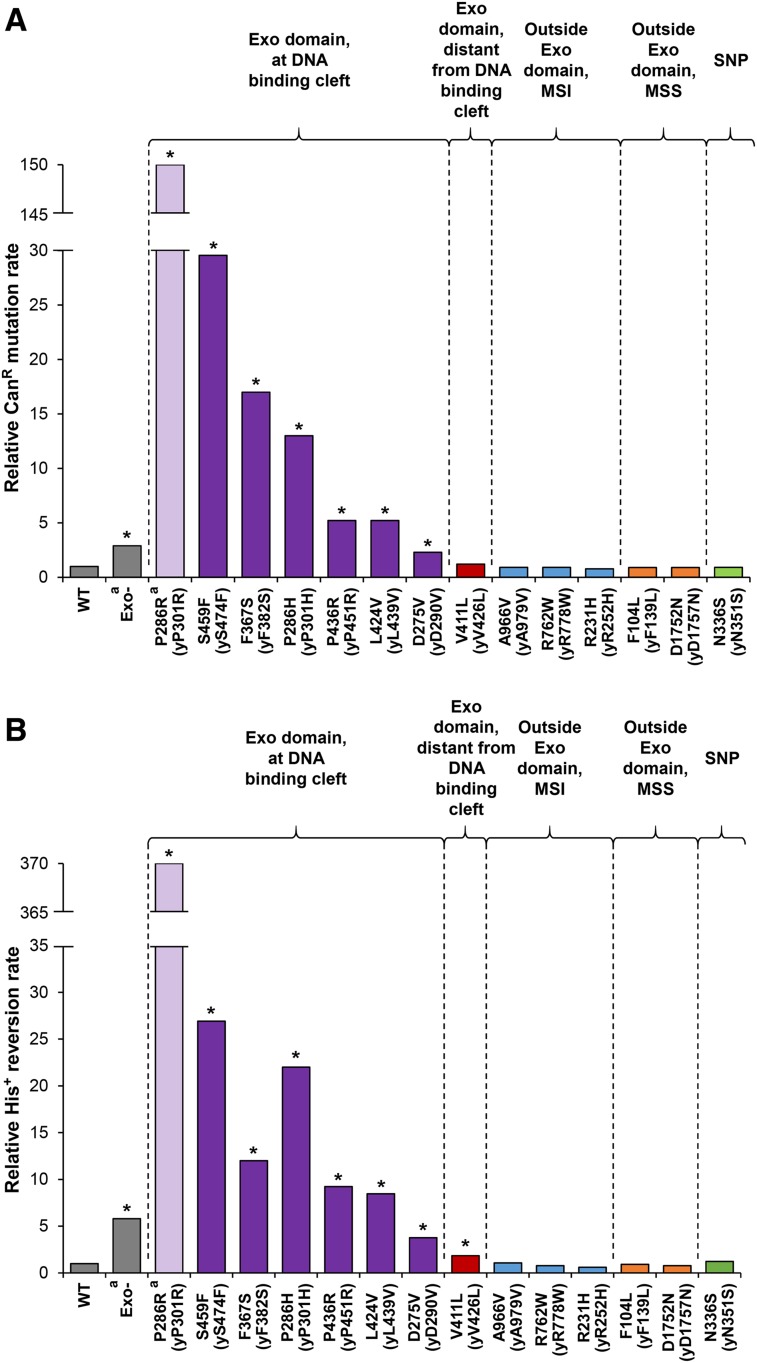
Mutator effects of cancer-associated Polε variants in haploid yeast strains. Mutation rates were measured in haploid strains in which the chromosomal *POL2* gene was replaced with mutant *pol2* alleles mimicking human *POLE* variants. The human variants are listed on the *x*-axis with the analogous yeast substitutions in parentheses. Exo^−^, exonuclease-deficient variant encoded by the *pol2-4* allele. (A) Can^R^ mutation relative to wild type (WT). (B) Reversion of the *his7-2* allele relative to WT. Data are from Table S3 in File S1. Asterisks indicate *P* < 0.05 by Wilcoxon–Mann–Whitney test compared with WT. ^a^Data from Kane and Shcherbakova (2014).

**Figure 3:**
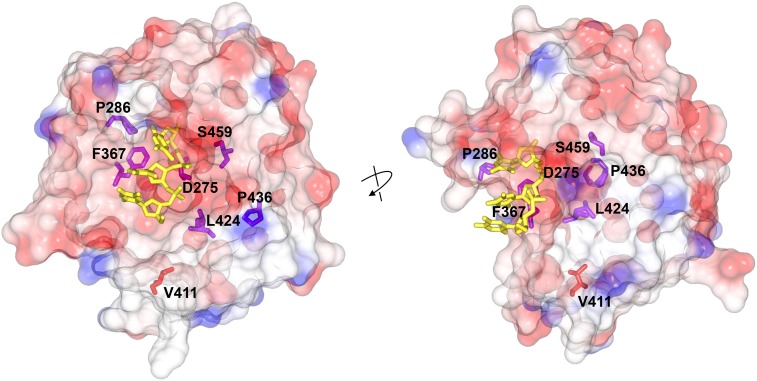
Location of cancer-associated Polε mutations within the exonuclease domain. Structure of the exonuclease domain of *Saccharomyces cerevisiae* Polε (PBD ID: 4M8O) is from Hogg *et al.* (2014). Single-stranded DNA (yellow) was modeled in the exonuclease active site by alignment with the structure of T4 polymerase–DNA complex (PBD ID: 1NOY) as done previously (Rayner *et al.* 2016). The protein surface is colored according to electrostatic potential, revealing the predominantly negatively charged exonuclease active site cleft. Two views of the same structure are shown, with the arrow indicating the approximate direction of rotation. Note the clustering of six cancer-associated mutations at the active site cleft (side chains shown in purple sticks) and the distant position of V411 (red sticks). The figure was generated with CCP4MG molecular graphics software.

We constructed haploid yeast strains with the corresponding chromosomal *pol2* alleles and measured the mutation rate using two primary assays. The Can^R^ forward mutation assay detects a variety of base substitutions, frameshifts, and complex mutations that deactivate the *CAN1* gene. The His^+^ reversion assay scores +1 events that revert the frameshift mutation *his7-2*, which is a single-base deletion in a run of eight As in the *HIS7* gene (Shcherbakova and Kunkel 1999). Most exonuclease domain variants had significant, although highly variable, mutator effects (Figure 2; purple bars). Although none were near the magnitude of the previously studied *pol2-P301R* allele (human *POLE-P286R* mimic; Kane and Shcherbakova 2014), the mutator effects still substantially exceeded the effect of the *pol2-4* (*pol2-D290A*,*E292A*) mutation, which completely inactivates Polε proofreading (Morrison *et al.* 1991) (Figure 2; Exo^−^). Thus, the vast majority of exonuclease domain variants must increase the mutation rate through mechanisms other than simple loss of exonuclease activity. One exception was *pol2-D290V* (mimicking human *POLE-D275V*), which had a rather weak mutator effect nearly identical to that of *pol2-4* (Figure 2). This was not completely unexpected, since *pol2-D290V* eliminates one of the two catalytic carboxylates affected by the *pol2-4* mutation. The main consequence of the *pol2-D290V* variant may indeed be defective proofreading. The only exonuclease domain variant that showed no significant increase in Can^R^ mutation and only marginally increased His^+^ reversion was *pol2-V426L*, mimicking human *POLE-V411L* (Figure 2; red bars). This was surprising, because V411L is the second most frequently observed variant after P286R, and it is clearly associated with high levels of hypermutation in tumors. While trying to explain the lack of a mutator effect in yeast, we noted that all variants showing mutator effects (Figure 2) are suggested by the crystal structure of Polε to directly alter the DNA binding cleft within the exonuclease domain (Hogg *et al.* 2014; Rayner *et al.* 2016; Figure 3). By contrast, V411L is located further away from the exonuclease active site (Figure 3), and neither the side chain of leucine nor that of valine at this position is expected to contact the DNA substrate. It has been reported previously that the mutational spectrum in V411L tumors is somewhat different from the spectrum in other *POLE*-mutant tumors (Church *et al.* 2013). Taken together, these observations suggest that the molecular mechanisms underlying the pathogenicity of V411L and DNA binding cleft mutations may not be the same (see *Discussion*).

The *pol2* mutations affecting amino acid residues outside the exonuclease domain and the *pol2-N351S* allele mimicking the SNP showed no mutator effect (Figure 2; blue, orange, and green bars). In addition to the *CAN1* and *his7-2* mutagenesis reporters, all strains also allowed for the measurement of *lys2*::*InsE_A14_* allele reversion, which scores −1 frameshifts in a run of 14 As, thus providing a readout for instability of mononucleotide repeats (Tran *et al.* 1997). Consistent with the lack of association between *POLE* mutations and an MSI phenotype in tumors, Lys^+^ reversion was barely, if at all, affected in the *pol2* mutants (Figure S1 in File S1).

### Mutator effects of DNA binding cleft variants correlate with their incidence in tumors

**Figure 4:**
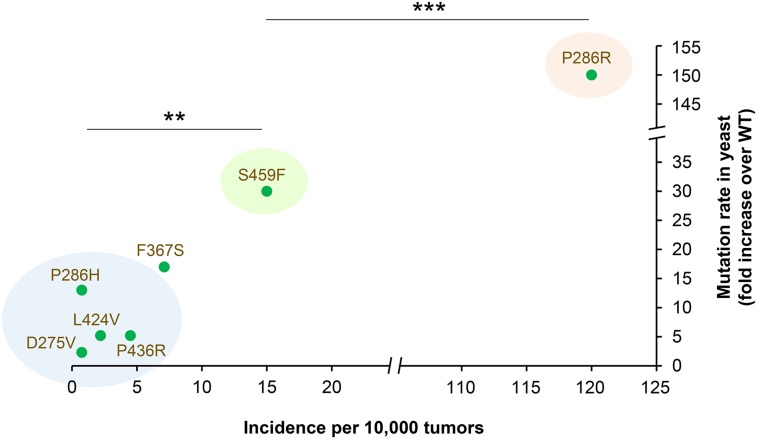
Relationship between the mutator effects of DNA binding cleft variants and their frequency in tumors. Relative Can^R^ mutation rates for the yeast analogs of cancer-associated variants (Figure 2 and Table S3 in File S1) were plotted against the frequency at which the corresponding variant has been reported in CRC and EC (Table S4 in File S1). The mutation rate for the yeast P286R mimic is from Kane and Shcherbakova (2014). The breaks in the *x* and *y* axes remove equal fractions of the two axes. Blue, green, and pink ovals highlight variants, for which statistical significance of differences in frequency are indicated by asterisks. **P < 0.01 for the frequency of S459F *vs.* frequency of each of the variants in the blue oval. ***P < 0.0001. The *P* values for pairwise comparisons of all variants are shown in Table S5 in File S1.

We hypothesized previously that the higher incidence of the P286R variant in cancers in comparison with other exonuclease domain changes is due to its exceptionally strong mutator effect and the resulting increased chances of accumulating cancer-driving mutations (Kane and Shcherbakova 2014). Indeed, the less common variants studied here were substantially weaker mutators (Figure 2). For the variants that caused a significant elevation of the mutation rate over wild-type levels (the DNA binding cleft variants), we further examined the relationship between their incidence in tumors and the mutator phenotype. We calculated the frequency of each variant using published sequencing data on >13,000 sporadic colorectal and endometrial tumors (Table S4 in File S1). Although the incidence estimates for the rarest variants are likely to be imprecise, in general, we observed that stronger mutator effects tended to be associated with higher variant frequency (Figure 4). The incidence of P286R was significantly higher than the incidence of the moderate mutator S459F, and the incidence of S459F significantly exceeded that of the weakest mutators P286H, P436R, L424V, and D275V. It is interesting that the correlation between the mutator effect and variant frequency was only observed for the DNA binding cleft variants. As described earlier, the very common but peripherally located V411L failed to produce any mutator effect in yeast, indicating that other factors must determine its high incidence.

The DNA binding cleft mutations are also known to variably affect the exonuclease activity of purified human Polε, ranging from a twofold reduction to a complete loss of exonuclease (Shinbrot *et al.* 2014). However, no correlation was seen between the extent of exonuclease deficiency or *in vitro* fidelity of Polε variants and their frequency in tumors (Figure S2 in File S1). These findings indicate that the preferential occurrence of some but not other DNA binding cleft variants in tumors is likely explained by the differences in the mutator effects *in vivo* and not by the extent of exonuclease deficiency. They also further confirm that the effects of exonuclease domain variants on the mutation rate are separable from their effects on proofreading.

### Mutator Polε variants are semidominant

**Figure 5:**
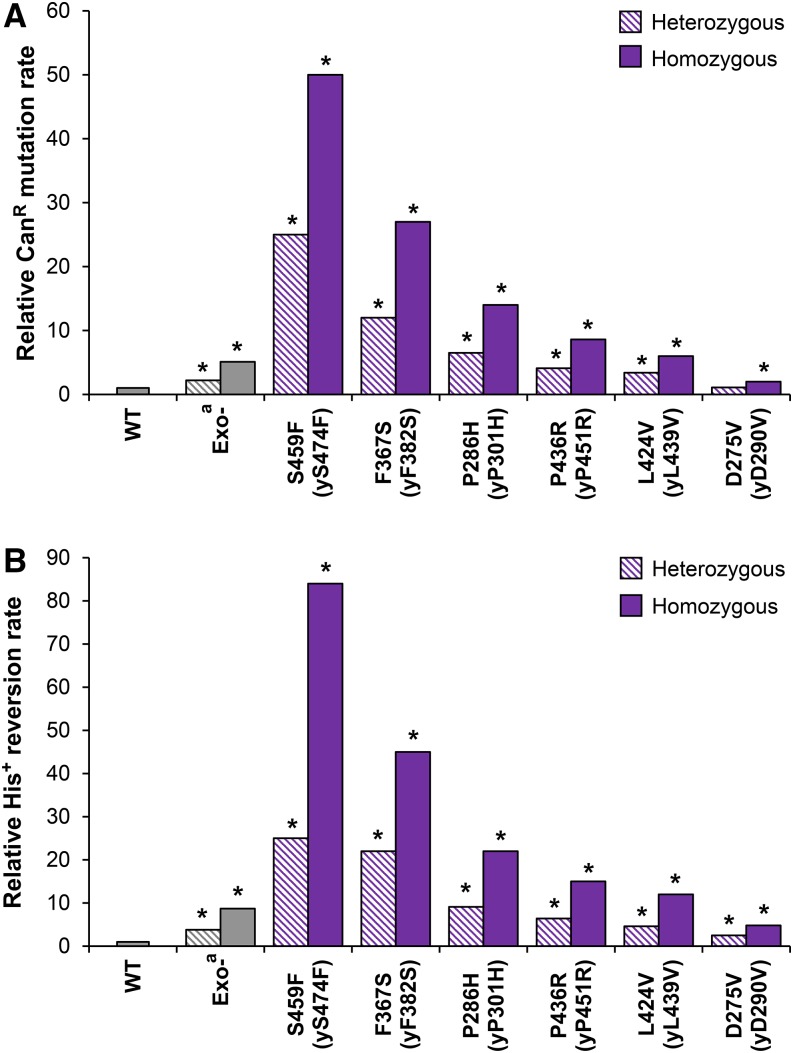
Mutator effects of cancer-associated Polε variants in diploid yeast strains. Mutation rates were measured in diploid yeast strains heterozygous or homozygous for *pol2* mutations mimicking the human *POLE* variants. Relative mutation rates are shown compared with wild type (WT). The human variants are listed on the *x*-axis with the analogous yeast substitutions in parentheses. Exo^−^, exonuclease-deficient variant encoded by the *pol2-4* allele. (A) Can^R^ mutation was measured in strains containing a single copy of the *CAN1* gene as described in *Materials and Methods*. (B) His^+^ reversion was measured in strains homozygous for the *his7-2* allele. Asterisks indicate *P* < 0.05 by Wilcoxon–Mann–Whitney test compared with WT. Data are from Table S6 in File S1. ^a^Data from Kane and Shcherbakova (2014).

Polε mutations are almost exclusively present in the heterozygous state in sporadic human tumors. Loss of heterozygosity (LOH) is also not required for carriers of germline *POLE* mutations to develop tumors (Palles *et al.* 2013). We found previously that heterozygosity for the *pol2-P301R* allele used to mimic the most common *POLE-P286R* variant confers a strong increase in mutagenesis, comparable to the effect of MMR deficiency (Kane and Shcherbakova 2014). However, it remained unclear whether the other, weaker mutator alleles could cause a substantial enough increase in mutagenesis when present in the heterozygous state. We constructed diploid strains heterozygous for the mutator *pol2* alleles and measured the rate of Can^R^ mutation and His^+^ reversion. All DNA binding cleft variants significantly increased the mutation rate at both reporter loci when present in the heterozygous state, with the exception of Can^R^ mutation in the weakest mutator strain *pol2-D290V* (Figure 5; hatched purple bars). These results further validate the pathogenicity of the majority of DNA binding cleft variants and explain why LOH is not necessary for hypermutability and tumorigenesis. Because LOH is still occasionally seen in both sporadic and hereditary cancers, and could potentially be associated with higher levels of hypermutation and earlier onset of the disease (Barbari and Shcherbakova 2017), we also investigated the impact of homozygosity of the mutator *pol2* alleles on mutagenesis. The mutation rate in homozygous diploid strains was approximately twice as high as that in the corresponding heterozygous strains (Figure 5; solid purple bars). Thus, the mutant and wild-type polymerases must equally contribute to DNA replication in the heterozygotes, and the LOH would likely further accelerate the accumulation of mutations and tumorigenesis in humans. Mutator effects of *pol2* alleles at the *lys2-InsE_A14_* homonucleotide repeat locus were weak, if present at all, similar to the results with haploids, but semidominance could still be noted in the cases where mutagenesis was significantly increased over the wild-type level (Figure S3 in File S1).

### Weak Polε mutators present in MMR^−^ tumors can act synergistically with MMR defects to increase mutation rate

**Figure 6:**
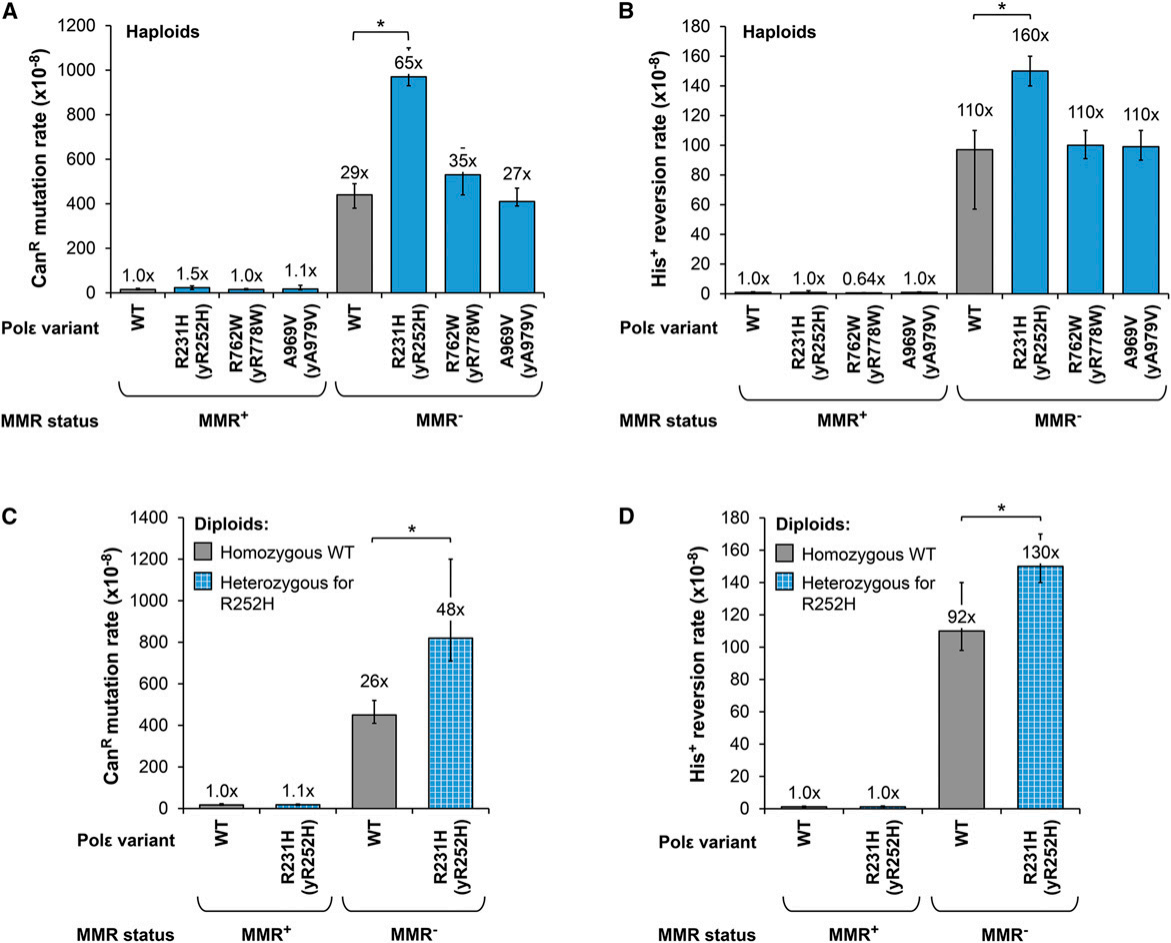
Effects of Polε variants found in MMR-deficient tumors on the mutation rate in MMR-deficient background. Mutation rates were measured in haploid (A and B) or diploid (C and D) strains containing chromosomal *pol2* mutations indicated on the *x*-axis. The MMR defect was mimicked by deleting the yeast *MLH1* gene. Mutation rates are given as the median for at least 18 independent cultures, with error bars indicating 95% confidence intervals. Fold increase in mutation rate relative to the strain with wild-type *POL2* and *MLH1* genes is shown above each bar. Asterisks indicate *P* < 0.05 by Wilcoxon–Mann–Whitney test. Data are from Table S7 in File S1.

Three *pol2* variants mimicking human *POLE* mutations reported in MMR-deficient MSI tumors had no mutator effect in MMR-proficient strains (Figure 2, blue bars). To determine whether these variants could make a meaningful contribution to genome instability in a MMR-deficient background, we measured their effects on the rate of Can^R^ mutation and His^+^ reversion in strains with a deletion of *MLH1*. This deletion mimics the MMR deficiency caused by the hypermethylation of the *MLH1* promoter, which is typically responsible for the MSI phenotype of tumors. The *pol2-R252H* variant caused a synergistic increase in the mutation rate in combination with the MMR defect. The synergy was observed both when *pol2-R252H* was present as the only *POL2* allele in haploids (Figure 6, A and B) and in the heterozygous state in diploids (Figure 6, C and D), suggesting a potentially significant role of the corresponding human variant when MMR is compromised. By contrast, the *pol2-R252H* allele caused no significant increase in the rate of *lys2-InsE_A14_* reversion beyond the expected dramatic increase conferred by the *MLH1* deletion (Figure S4 in File S1). These assays predict that the corresponding human variant, R231H, would not further increase MSI in the MMR-deficient tumor but could accelerate the accumulation of mutations in nonrepetitive sequences. The other two alleles mimicking *POLE* variants R762W and A966V found in MSI tumors had no effect on mutation rate at any of the three reporter loci in the MMR-deficient background (Figure 6, A and B and Figure S4A in File S1). Therefore, it is probable that these two variants are neutral passenger mutations that arose as a consequence of the hypermutation caused by defective MMR. Taken together, these results suggest that although many of the *POLE* mutations in MMR-deficient tumors may have no functional significance, some could result in mildly error-prone polymerase variants that can act synergistically with faulty MMR to further elevate mutagenesis.

## Discussion

Of the hundreds of somatic Polε mutations reported in tumors to date, the functional impact *in vivo* has only been assessed for the most frequently recurring Polε-P286R variant (Kane and Shcherbakova 2014). To gain a comprehensive understanding of the consequences of these mutations, we set out to characterize 13 additional cancer-associated Polε variants in the yeast model system. We show that amino acid substitutions near the DNA binding cleft in the exonuclease domain (the yeast mimics of D275V, P286H, F367S, L424V, P436R, and S459F) increase mutagenesis (Figure 2 and Figure 5). The increased mutation rate strongly argues that these variants drive the genomic instability in tumors, as previous *in silico* and *in vitro* analyses suggested (Church *et al.* 2013; Palles *et al.* 2013; Shinbrot *et al.* 2014; Rayner *et al.* 2016; Campbell *et al.* 2017). The magnitude of the mutator effect is highly variable and correlates with the incidence of these variants in tumors (Figure 4). This correlation is consistent with the idea that the chance of any particular *POLE* mutant cell lineage developing into a tumor (and, thus, the chance of the *POLE* mutation being detected by sequencing of tumor samples) is proportional to the severity of the mutator phenotype. Although different DNA binding cleft variants could also conceivably arise at different rates, our data suggest that the cancer risk conferred by each variant is a major determinant of its ultimate frequency in tumors.

The magnitude of the mutator effects we observed argues against the common view that the cancer-associated Polε mutations act by disabling proofreading. With the exception of the mutation affecting the catalytic aspartic acid residue (*pol2-D290V* mimicking human *POLE-D275V*), all other DNA binding cleft variants increased the mutation rate more than the *pol2-4* mutation that completely eliminates proofreading (Figure 2). Most of these variants only partially reduce the exonuclease activity of human Polε *in vitro* (Shinbrot *et al.* 2014); therefore, if this was the only consequence at the protein level, the yeast mimics would be expected to be weaker mutators than *pol2-4*, not stronger. The disconnect between the exonuclease defect and the mutator phenotype is further apparent from the observation that the frequency of DNA binding cleft variants in tumors does not correlate with the degree of proofreading impairment (Figure S2 in File S1), whereas it does correlate with the mutator effect *in vivo* (Figure 4). Thus, the mutations must impact the polymerase in some additional way(s). It is likely that the pathogenicity of DNA binding cleft mutations is determined by these additional defects, and not by the loss of proofreading *per se*. The exact nature of these defects remains to be investigated.

The rather mild mutator effect of the yeast analog of *POLE-L424V* (Figure 2) deserves a special comment. Although it is consistent with the relatively low frequency of this variant in sporadic tumors, L424V is the most frequent germline DNA polymerase variant in patients with hereditary CRC. It has been reported in >20 families (Palles *et al.* 2013; Valle *et al.* 2014; Chubb *et al.* 2015; Elsayed *et al.* 2015; Spier *et al.* 2015) and has also been observed as a *de novo* germline variant in some CRC cases (Valle *et al.* 2014; Elsayed *et al.* 2015). The high prevalence of this specific variant in hereditary CRC was puzzling. It could be argued that stronger mutators escaped detection because they do not result in the specific clinical syndrome—a high-penetrance predisposition to colorectal adenomas and carcinomas—in which L424V is implicated. However, other modest mutators of the same range as L424V have not been detected in hereditary cancers either. A possible clue is offered by the genomic DNA sequence context of the mutation site, which likely represents a mutational hotspot (Barbari and Shcherbakova 2017). The mutation is a C to G transversion occurring in close proximity to a 10-nt GC-rich palindromic sequence that can form a hairpin-type structure [Figure 3 in Barbari and Shcherbakova (2017)]. We have shown previously that such short hairpins impede DNA synthesis by replicative DNA polymerases and promote mutations in the nearby region, particularly C→G transversions (Northam *et al.* 2014). The mechanism of these mutations involves the recruitment of translesion synthesis DNA polymerases that bypass the replication-blocking structures in an error-prone manner. Thus, it is likely that the *POLE-L424V* variant is seen frequently in cancers not because it is more pathogenic than other *POLE* mutations, but because it is generated more frequently at the DNA level, either during normal DNA replication or during various stress responses. The exceptionally high recurrence of L424V as a germline variant, but not as much as a somatic one, suggests an intriguing possibility that the bypass of small hairpin structures is more problematic in germline cells.

Unexpected based on the known properties of *POLE* mutant tumors was the absence of a readily detectable mutator effect of the *POLE-V411L* analog. V411L is one of the two most frequent variants in hypermutated sporadic tumors. A recent study of mutation burden in >81,000 tumors, the largest collection yet, confirmed its invariable association with hypermutation (Campbell *et al.* 2017). From a mechanistic point of view, the lack of striking phenotypic consequences of this conservative amino acid substitution is not overly surprising. Human Polε-V411L showed only a threefold reduction in exonuclease activity *in vitro* compared with the wild-type Polε, in contrast to an up to 20-fold decrease in some other cancer-associated variants (Shinbrot *et al.* 2014). Also, because V411 is located further away from the DNA binding interface (Figure 3), this mutation may not cause that additional unidentified change in properties that makes the other exonuclease domain variants strong mutators. If the mild exonuclease defect is the only consequence of the V411L substitution, it may not produce a statistically significant increase in mutagenesis in our yeast assays. Although this provides a plausible explanation for the lack of a mutator effect, the reasons for the high recurrence of this variant in tumors remain unclear. One possibility is that even a very weak mutator phenotype may be sufficient to accumulate a large number of mutations over many years. The high prevalence could then be explained if this genomic site behaved as a mutational hotspot, similar to the L424V case. We could not immediately pinpoint any specific DNA sequence features around the mutation site that would support the hotspot hypothesis. A recent study also suggested that cells with the V411L mutation may, in fact, take a shorter path to cancer: a germline V411L variant was identified in a pediatric CRC case with an onset at least 10 yr earlier than is typical for carriers of other *POLE* mutations (Wimmer *et al.* 2017). An alternative explanation is that the V411L substitution causes a defect in a protein–protein interaction important for replication fidelity that cannot be modeled in yeast. It is also possible that expression of the mutator phenotype by V411L cells requires some additional factors that are not present during growth under standard laboratory conditions, such as exposure to DNA-damaging agents or replication stress. Although highly speculative, a fourth possibility is that the V411L variant is not the primary driver of hypermutability in tumors but arises concomitantly with another genome maintenance defect. As mentioned previously, the spectrum of mutations accumulating in tumors with the V411L variant differs somewhat from the mutational specificity of other *POLE* mutant tumors (Church *et al.* 2013), suggesting different mutagenesis mechanisms. Further studies are needed to determine the reasons for the pathogenicity of this atypical variant and its high recurrence, including analysis in human cells to assess potential consequences that may not be detected in yeast.

## Supplementary Material

Supplemental material is available online at www.g3journal.org/lookup/suppl/doi:10.1534/g3.118.200042/-/DC1.
