# Supplementary material for: Functional Analysis of Cancer-Associated DNA Polymerase ε Variants in Saccharomyces cerevisiae
Source: G3 (Bethesda). 2018 Jan 19;8(3):1019–29. doi: 10.1534/g3.118.200042 (PMC5844290; doi:10.1534/g3.118.200042)
Supplement: Supplementary file 1 [file 1019FileS1.pdf]

SUPPLEMENTARY MATERIAL

Functional analysis of cancer-associated DNA polymerase ε variants in *Saccharomyces cerevisiae*

Stephanie R. Barbari, Daniel P. Kane[1], Elizabeth A. Moore and Polina V. Shcherbakova

Eppley Institute for Research in Cancer and Allied Diseases, Fred & Pamela Buffett Cancer Center, University of Nebraska Medical Center, Omaha, NE 68198, USA

[1] *Present address:* Daniel P. Kane, Department of Biological and Environmental Sciences, Le Moyne College, Syracuse, NY 13214

Table S1 Genotypes of yeast strains used for construction of *pol2-x* mutants and mutation rate measurements

Strain	Relevant chromosomal mutation	Genotype
**Haploid strains**		
TM30	WT	*MATa ade5-1 lys2-Tn5-13 trp1-289 his7-2 leu2-3,112 ura3-4 CAN1::LEU2*
TM44	WT	*MATα ade5-1 lys2-InsE$_{A14}$ trp1-289 his7-2 leu2-3,112 ura3-52 can1Δ::loxP*
DK039a/DK039b	*pol2-F139L*	*MATa ade5-1 lys2-InsE$_{A14}$ trp1-289 his7-2 leu2-3,112 ura3-X CAN1::LEU2 pol2-F139L*
SB042/SB044	*pol2-R252H*	*MATa ade5-1 lys2-Tn5-13 trp1-289 his7-2 leu2-3,112 ura3-4 CAN1::LEU2 pol2-R252H*
SB046/SB051	*pol2-R252H*	*MATα ade5-1 lys2-InsE$_{A14}$ trp1-289 his7-2 leu2-3,112 ura3-52 can1Δ::loxP pol2-R252H*
SI022/SI023	*pol2-D290V*	*MATa ade5-1 lys2-Tn5-13 trp1-289 his7-2 leu2-3,112 ura3-4 CAN1::LEU2 pol2-D290V*
SI024/SI026	*pol2-D290V*	*MATα ade5-1 lys2-InsE$_{A14}$ trp1-289 his7-2 leu2-3,112 ura3-52 can1Δ::loxP pol2-D290V*
DK046/DK047	*pol2-P301H*	*MATα ade5-1 lys2-InsE$_{A14}$ trp1-289 his7-2 leu2-3,112 ura3-X CAN1::LEU2 pol2-P301H*
SB112/SB113	*pol2-P301H*	*MATa ade5-1 lys2-InsE$_{A14}$ trp1-289 his7-2 leu2-3,112 ura3-X can1Δ::loxP pol2-P301H*
DK048	*pol2-F382S*	*MATα ade5-1 lys2-Tn5-13 trp1-289 his7-2 leu2-3,112 ura3-X can1Δ::loxP pol2-F382S*
DK049/DK050	*pol2-F382S*	*MATa ade5-1 lys2-InsE$_{A14}$ trp1-289 his7-2 leu2-3,112 ura3-X CAN1::LEU2 pol2-F382S*
SB052/SB053	*pol2-V426L*	*MATα ade5-1 lys2-InsE$_{A14}$ trp1-289 his7-2 leu2-3,112 ura3-52 can1Δ::loxP pol2-V426L*
SB054/SB055	*pol2-V426L*	*MATα ade5-1 lys2-InsE$_{A14}$ trp1-289 his7-2 leu2-3,112 ura3-52 can1Δ::loxP pol2-V426L*
SI030/SI031	*pol2-L439V*	*MATa ade5-1 lys2-Tn5-13 trp1-289 his7-2 leu2-3,112 ura3-4 CAN1::LEU2 pol2-L439V*
SI032/SI033	*pol2-L439V*	*MATα ade5-1 lys2-InsE$_{A14}$ trp1-289 his7-2 leu2-3,112 ura3-52 can1Δ::loxP pol2-L439V*
SB063/SB065	*pol2-P451R*	*MATa ade5-1 lys2-Tn5-13 trp1-289 his7-2 leu2-3,112 ura3-4 CAN1::LEU2 pol2-P451R*
SB066/SB068	*pol2-P451R*	*MATα ade5-1 lys2-InsE$_{A14}$ trp1-289 his7-2 leu2-3,112 ura3-52 can1Δ::loxP pol2-P451R*
SB069/SB071	*pol2-S474F*	*MATa ade5-1 lys2-Tn5-13 trp1-289 his7-2 leu2-3,112 ura3-4 CAN1::LEU2 pol2-S474F*
SB073/SB074	*pol2-S474F*	*MATα ade5-1 lys2-InsE$_{A14}$ trp1-289 his7-2 leu2-3,112 ura3-52 can1Δ::loxP pol2-S474F*

Table S1 Genotypes of yeast strains used for construction of *pol2-x* mutants and mutation rate measurements (Continued)

Strain	Relevant chromosomal mutation	Genotype
DK051a/DK051b	*pol2-R778W*	*MATα ade5-1 lys2-InsE$_{A14}$ trp1-289 his7-2 leu2-3,112 ura3-X CAN1::LEU2 pol2-R778W*
SB075/SB077	*pol2-A979V*	*MATa ade5-1 lys2-Tn5-13 trp1-289 his7-2 leu2-3,112 ura3-4 CAN1::LEU2 pol2-A979V*
SB079/SB081	*pol2-A979V*	*MATα ade5-1 lys2-InsE$_{A14}$ trp1-289 his7-2 leu2-3,112 ura3-52 can1Δ::loxP pol2-A979V*
DK055a/DK055b	*pol2-D1757N*	*MATa ade5-1 lys2-InsE$_{A14}$ trp1-289 his7-2 leu2-3,112 ura3-X CAN1::LEU2 pol2-D1757N*
SB083/SB084	*mlh1Δ*	*MATa ade5-1 lys2-Tn5-13 trp1-289 his7-2 leu2-3,112 ura3-4 CAN1::LEU2 mlh1Δ::hphMX4*
SB086/SB087	*mlh1Δ*	*MATα ade5-1 lys2-InsE$_{A14}$ trp1-289 his7-2 leu2-3,112 ura3-52 can1Δ::loxP mlh1Δ::hphMX4*
SB088/SB089/ SB090/SB091	*pol2-R252H mlh1Δ*	*MATa ade5-1 lys2-Tn5-13 trp1-289 his7-2 leu2-3,112 ura3-4 CAN1::LEU2 pol2-R252H mlh1Δ::hphMX4*
SB092/SB093/ SB094	*pol2-R252H mlh1Δ*	*MATα ade5-1 lys2-InsE$_{A14}$ trp1-289 his7-2 leu2-3,112 ura3-52 can1Δ::loxP pol2-R252H mlh1Δ::hphMX4*
SI003/SI004/ SI005/SI006	*pol2-R778W mlh1Δ*	*MATα ade5-1 lys2-InsE$_{A14}$trp1-289 his7-2 leu2-3,112 ura3-X CAN1::LEU2 pol2-R778W mlh1Δ::hphMX4*
SB095/SB096/ SB097/SB098	*pol2-A979V mlh1Δ*	*MATa ade5-1 lys2-Tn5-13 trp1-289 his7-2 leu2-3,112 ura3-4 CAN1::LEU2 pol2-A979V mlh1Δ::hphMX4*
SB099/SB100/ SB101/SB102	*pol2-A979V mlh1Δ*	*MATα ade5-1 lys2-InsE$_{A14}$ trp1-289 his7-2 leu2-3,112 ura3-52 can1Δ::loxP pol2-A979V mlh1Δ::hphMX4*
**Diploid strains**		
TM63/SB539/ SB540	WT	*MATa/MATα ade5-1/ade5-1 lys2-Tn5-13/lys2-InsE$_{A14}$ trp1-289/trp1-289 his7-2/his7-2 leu2-3,112/leu2-3,112 ura3-4/ura3-52 CAN1::LEU2/can1Δ::loxP*
SB555/SB556	*pol2-R252H/POL2*	*MATa/MATα ade5-1/ade5-1 lys2-Tn5-13/lys2-InsE$_{A14}$ trp1-289/trp1-289 his7-2/his7-2 leu2-3,112/leu2-3,112 ura3-4/ura3-52 CAN1::LEU2/can1Δ::loxP pol2-R252H/POL2*
SI531/SI533	*pol2-D290V/POL2*	*MATa/MATα ade5-1/ade5-1 lys2-Tn5-13/lys2-InsE$_{A14}$ trp1-289/trp1-289 his7-2/his7-2 leu2-3,112/leu2-3,112 ura3-4/ura3-52 CAN1::LEU2/can1Δ::loxP pol2-D290V/POL2*

Table S1 Genotypes of yeast strains used for construction of *pol2-x* mutants and mutation rate measurements (Continued)

Strain	Relevant chromosomal mutation	Genotype
SI527/SI528/ SI529/SI530	*pol2-D290V/pol2-D290V*	*MATa/MATα ade5-1/ade5-1 lys2-Tn5-13/lys2-InsE$_{A14}$ trp1-289/trp1-289 his7-2/his7-2 leu2-3,112/leu2-3,112 ura3-4/ura3-52 CAN1::LEU2/can1Δ::loxP pol2-D290V/pol2-D290V*
SI505/SI507	*pol2-P301H/POL2*	*MATa/MATα ade5-1/ade5-1 lys2-InsE$_{A14}$/lys2-InsE$_{A14}$ trp1-289/trp1-289 his7-2/his7-2 leu2-3,112/leu2-3,112 ura3-X/ura3-X CAN1::LEU2/can1Δ::loxP pol2-P301H/POL2*
SB557/SB558/ SB559	*pol2-P301H/pol2-P301H*	*MATa/MATα ade5-1/ade5-1 lys2::InsE$_{A14}$/lys2::InsE$_{A14}$ trp1-289/trp1-289 his7-2/his7-2 leu2-3,112/leu2-3,112 ura3-X/ura3-X CAN1::LEU2/can1Δ::loxP pol2-P301H/pol2-P301H*
SI509/SI511	*pol2-F382S/POL2*	*MATa/MATα ade5-1/ade5-1 lys2-InsE$_{A14}$/lys2-InsE$_{A14}$ trp1-289/trp1-289 his7-2/his7-2 leu2-3,112/leu2-3,112 ura3-X/ura3-52 CAN1::LEU2/can1Δ::loxP pol2-F382S/POL2*
SI514/SI516	*pol2-F382S/pol2-F382S*	*MATa/MATα ade5-1/ade5-1 lys2-InsE$_{A14}$/lys2-Tn5-13 trp1-289/trp1-289 his7-2/his7-2 leu2-3,112/leu2-3,112 ura3-X/ura3-X CAN1::LEU2/can1Δ::loxP pol2-F382S/pol2-F382S*
SI522/SI526	*pol2-L439V/POL2*	*MATa/MATα ade5-1/ade5-1 lys2-Tn5-13/lys2-InsE$_{A14}$ trp1-289/trp1-289 his7-2/his7-2 leu2-3,112/leu2-3,112 ura3-4/ura3-52 CAN1::LEU2/can1Δ::loxP pol2-L439V/POL2*
SI518/SI519/ SI520/SI521	*pol2-L439V/pol2-L439V*	*MATa/MATα ade5-1/ade5-1 lys2-Tn5-13/lys2-InsE$_{A14}$ trp1-289/trp1-289 his7-2/his7-2 leu2-3,112/leu2-3,112 ura3-4/ura3-52 CAN1::LEU2/can1Δ::loxP pol2-L439V/pol2-L439V*
SB542/SB545	*pol2-P451R/POL2*	*MATa/MATα ade5-1/ade5-1 lys2-Tn5-13/lys2-InsE$_{A14}$ trp1-289/trp1-289 his7-2/his7-2 leu2-3,112/leu2-3,112 ura3-4/ura3-52 CAN1::LEU2/can1Δ::loxP pol2-P451R/POL2*
SB547/SB548	*pol2-P451R/pol2-P451R*	*MATa/MATα ade5-1/ade5-1 lys2-Tn5-13/lys2-InsE$_{A14}$ trp1-289/trp1-289 his7-2/his7-2 leu2-3,112/leu2-3,112 ura3-4/ura3-52 CAN1::LEU2/can1Δ::loxP pol2-P451R/pol2-P451R*
SB549/SB551	*pol2-S474F/POL2*	*MATa/MATα ade5-1/ade5-1 lys2-Tn5-13/lys2-InsE$_{A14}$ trp1-289/trp1-289 his7-2/his7-2 leu2-3,112/leu2-3,112 ura3-4/ura3-52 CAN1::LEU2/can1Δ::loxP pol2-S474F/POL2*
SB552/SB553	*pol2-S474F/pol2-S474F*	*MATa/MATα ade5-1/ade5-1 lys2-Tn5-13/lys2-InsE$_{A14}$ trp1-289/trp1-289 his7-2/his7-2 leu2-3,112/leu2-3,112 ura3-4/ura3-52 CAN1::LEU2/can1Δ::loxP pol2-S474F/pol2-S474F*
SB560/SB561	*mlh1Δ::hphMX4/mlh1Δ:: hphMX4*	*MATa/MATα ade5-1/ade5-1 lys2-Tn5-13/lys2-InsE$_{A14}$ trp1-289/trp1-289 his7-2/his7-2 leu2-3,112/leu2-3,112 ura3-4/ura3-52 CAN1::LEU2/can1Δ::loxP mlh1Δ::hphMX4/mlh1Δ::hphMX4*
SB562/SB563	*pol2-R252H/POL2 mlh1Δ::hphMX4/mlh1Δ:: hphMX4*	*MATa/MATα ade5-1/ade5-1 lys2-Tn5-13/lys2-InsE$_{A14}$ trp1-289/trp1-289 his7-2/his7-2 leu2-3,112/leu2-3,112 ura3-4/ura3-52 CAN1::LEU2/can1Δ::loxP pol2-R252H/POL2 mlh1Δ::hphMX4/mlh1Δ::hphMX4*

Table S2 Plasmid, restriction site, and method used to construct *pol2* mutants

Human POLE variant	Yeast Pol2 mimic	Plasmid	Restriction site	Mutation in truncated or full-length *POL2* after integration	Construction method[a]
F104L	F139L	YIpDK1	*Bam*HI	Full-length	1
R231H	R252H	YIpDK1	*Bgl*II	Truncated	2
D275V	D290V	YIpDK1	*Bam*HI	Full-length	1
P286H	P301H	YIpDK1	*Bam*HI	Full-length	1
N336S	N351S	YIpDK1	*Bam*HI	Full-length	1
F367S	F382S	YIpDK1	*Bam*HI	Full-length	1
V411L	V426L	YIpDK1	*Bgl*II	Truncated	2
L424V	L439V	YIpDK1	*Bam*HI	Full-length	1
P436R	P451R	YIpDK1	*Bgl*II	Truncated	2
S459F	S474F	YIpDK1	*Bgl*II	Truncated	2
R762W	R778W	p173	*Age*I	Full-length	1
A966V	A979V	p173	*Bsr*GI	Truncated	2
D1752N	D1757N	p174	*Age*I	Full-length	1

[a]Refers to the two methods described in the Materials and Methods section.

Table S3 Spontaneous mutation rates in haploid yeast strains with *pol2* mutations mimicking human *POLE* variants

Human POLE variant	Yeast *POL2* allele	Can^R mutation		His^+ reversion		Lys^+ reversion	
		Mutation rate ($x10^{-8}$)[a]	Fold increase	Mutation rate ($x10^{-8}$)[a]	Fold increase	Mutation rate ($x10^{-8}$)[a]	Fold increase
WT	*POL2*	23 (17-32)	1.0	1.3 (0.76-1.9)	1.0	31 (25-49)	1.0
F104L	*pol2-F139L*	21 (18-23)	0.91	1.2 (0.94-1.5)	0.92	26 (24-34)	0.84
R231H	*pol2-R252H*	18 (16-21)	0.78	0.78 (0.58-0.95)	0.60	36 (33-46)	1.2
D275V	*pol2-D290V*	**53 (44-55)**	**2.3**	**4.9 (4.2-5.9)**	**3.8**	**42 (36-44)**	**1.4**
P286H	*pol2-P301H*	**290 (250-440)**	**13**	**29 (25-37)**	**22**	**51 (40-63)**	**1.6**
N336S	*pol2-N351S*	21 (19-25)	0.91	1.6 (1.3-2.4)	1.2	32 (30-36)	1.0
F367S	*pol2-F382S*	**380 (300-490)**	**17**	**16 (8.7-22)**	**12**	56 (30-140)	1.8
V411L	*pol2-V426L*	28 (25-32)	1.2	**2.4 (2.0-3.3)**	**1.8**	30 (28-35)	0.97
L424V	*pol2-L439V*	**120 (82-140)**	**5.2**	**11 (9.6-16)**	**8.5**	32 (27-36)	1.0
P436R	*pol2-P451R*	**120 (98-150)**	**5.2**	**12 (11-16)**	**9.2**	**40 (36-49)**	**1.3**
S459F	*pol2-S474F*	**680 (560-800)**	**30**	**35 (30-47)**	**27**	**71 (62-87)**	**2.3**
R762W	*pol2-R778W*	21 (17-25)	0.91	1.0 (0.59-1.6)	0.77	26 (22-29)	0.84
A969V	*pol2-A979V*	21 (19-28)	0.91	1.4 (0.87-1.7)	1.1	31 (27-34)	1.0
D1752N	*pol2-D1757N*	21 (15-32)	0.91	1.0 (0.78-1.4)	0.77	22 (20-27)	0.71

[a]Mutation rates are given as the median of at least 18 independent cultures, with 95% confidence intervals in parentheses. Fold increase is shown relative to the wild-type haploid (WT). Bold text indicates $p < 0.05$ by Wilcoxon-Mann-Whitney compared to WT. Data from this table were used to generate Figure 2.

Table S4 Summary of published tumor sequencing data used to calculate the frequency of DNA binding cleft variants

Human *POLE* variant	Exon	No. times reported[a]	No. tumors analyzed[b]	Incidence per 10,000 tumors
D275V	9	1	13,283	0.75
P286H	9	1	13,283	0.75
P286R	9	160	13,159	120
F367S	11	5	7,079	7.1
L424V	13	3	13,424	2.2
P436R	13	6	13,424	4.5
S459F	14	18	11,831	15

[a]Number of times the variant has been reported in sporadic CRC and EC.
[b]Total number of tumors from studies where the corresponding exon was sequenced.

References

Ahn SM, Ansari AA, Kim J, Kim D, Chun SM, Kim TW*, et al.* The somatic *POLE* P286R mutation defines a unique subclass of colorectal cancer featuring hypermutation, representing a potential genomic biomarker for immunotherapy. *Oncotarget* 2016; 7*:* 68638-68649.

Bellone S, Centritto F, Black J, Schwab C, English D, Cocco E*, et al.* Polymerase ε (POLE) ultra-mutated tumors induce robust tumor-specific CD4+ T cell responses in endometrial cancer patients. *Gynecol Oncol* 2015; 138*:* 11-17.

Billingsley CC, Cohn DE, Mutch DG, Stephens JA, Suarez AA, and Goodfellow PJ. Polymerase ε (*POLE*) mutations in endometrial cancer: clinical outcomes and implications for Lynch syndrome testing. *Cancer* 2015; 121*:* 386-394.

Cancer Genome Atlas Network. Comprehensive molecular characterization of human colon and rectal cancer. *Nature* 2012; 487: 330-337.

Cancer Genome Atlas Network. Integrated genomic characterization of endometrial carcinoma. *Nature* 2013; 497: 67-73.

Church DN, Briggs SE, Palles C, Domingo E, Kearsey SJ, Grimes JM*, et al.* DNA polymerase ε and δ exonuclease domain mutations in endometrial cancer. *Hum Mol Genet* 2013; 22*:* 2820-2828.

Church DN, Stelloo E, Nout RA, Valtcheva N, Depreeuw J, ter Haar N*, et al.* Prognostic significance of *POLE* proofreading mutations in endometrial cancer. *J Natl Cancer Inst* 2015; 107*:* 402.

Domingo E, Freeman-Mills L, Rayner E, Glaire M, Briggs S, Vermeulen L*, et al.* Somatic *POLE* proofreading domain mutation, immune response, and prognosis in colorectal cancer: a retrospective, pooled biomarker study. *Lancet Gastroenterol Hepatol* 2016; 1*:* 207-216.

Espinosa I, D'Angelo E, Palacios J, and Prat J. Mixed and ambiguous endometrial carcinomas: A heterogenous group of tumors with different clinicopathologic and molecular genetic features. *Am J Surg Pathol* 2016; 40: 972-981.

Espinosa I, Lee CH, D'Angelo E, Palacios J, and Prat J. Undifferentiated and dedifferentiated endometrial carcinomas with pole exonuclease domain mutations have a favorable prognosis. *Am J Surg Pathol* 2017; 41: 1121-1128.

Giannakis M, Mu XJ, Shukla SA, Qian ZR, Cohen O, Nishihara R*, et al.* Genomic correlates of immune-cell infiltrates in colorectal carcinoma. *Cell Rep* 2016; 17*:* 1206.

Grossman RL, Heath AP, Ferretti V, Varmus HE, Lowy DR, Kibbe WA, et al. Toward a shared vision for cancer genomic data. *N Engl J Med* 2016; 375: 1109-1112; https://portal.gdc.cancer.gov/ [Last accessed 11/10/17].

Jesinghaus M, Pfarr N, Endris V, Kloor M, Volckmar AL, Brandt R*, et al.* Genotyping of colorectal cancer for cancer precision medicine: Results from the IPH Center for Molecular Pathology. *Genes Chromosomes Cancer* 2016; 55*:* 505-521.

Kane DP and Shcherbakova PV. A common cancer-associated DNA polymerase ε mutation causes an exceptionally strong mutator phenotype, indicating fidelity defects distinct from loss of proofreading. *Cancer Res* 2014; 74*:* 1895-1901.

Köbel M, Meng B, Hoang LN, Almadani N, Li X, Soslow RA*, et al.* Molecular analysis of mixed endometrial carcinomas shows clonality in most cases. *Am J Surg Pathol* 2016; 40*:* 166-180.

Kothari N, Teer JK, Abbott AM, Srikumar T, Zhang Y, Yoder SJ*, et al.* Increased incidence of *FBXW7* and *POLE* proofreading domain mutations in young adult colorectal cancers. *Cancer* 2016; 122*:* 2828-2835.

Le Gallo M, O'Hara AJ, Rudd ML, Urick ME, Hansen NF, O'Neil NJ*, et al.* Exome sequencing of serous endometrial tumors identifies recurrent somatic mutations in chromatin-remodeling and ubiquitin ligase complex genes. *Nat Genet* 2012; 44*:* 1310-1315.

McConechy MK, Talhouk A, Leung S, Chiu D, Yang W, Senz J, et al. Endometrial carcinomas with *POLE* exonuclease domain mutations have a favorable prognosis. *Clin Cancer Res* 2016; 22: 2865-2873.

Meng B, Hoang LN, McIntyre JB, Duggan MA, Nelson GS, Lee CH*, et al. POLE* exonuclease domain mutation predicts long progression-free survival in grade 3 endometrioid carcinoma of the endometrium. *Gynecol Oncol* 2014; 134*:* 15-19.

Nowak JA, Yurgelun MB, Bruce JL, Rojas-Rudilla V, Hall DL, Shivdasani P*, et al.* Detection of mismatch repair deficiency and microsatellite instability in colorectal adenocarcinoma by targeted next-generation sequencing. *J Mol Diagn* 2017; 19*:* 84-91

Rosa-Rosa JM, Leskelä S, Cristóbal-Lana E, Santón A, López-García MÁ, Muñoz G, et al. Molecular genetic heterogeneity in undifferentiated endometrial carcinomas. *Mod Pathol* 2016; 29: 1390-1398.

Seshagiri S, Stawiski EW, Durinck S, Modrusan Z, Storm EE, Conboy CB, et al. Recurrent R-spondin fusions in colon cancer. Nature 2012; 488: 660-664.

Shinbrot E, Henninger EE, Weinhold N, Covington KR, Schultz N, Chao H*, et al.* Exonuclease mutations in DNA polymerase ε reveal replication strand specific mutation patterns and human origins of replication. *Genome Res* 2014; 24*:* 1740-1750.

Stadler ZK, Battaglin F, Middha S, Hechtman JF, Tran C, Cercek A*, et al.* Reliable detection of mismatch repair deficiency in colorectal cancers using mutational load in next-generation sequencing panels. *J Clin Oncol* 2016; 34*:* 2141-2147

Stenzinger A, Pfarr N, Endris V, Penzel R, Jansen L, Wolf T*, et al.* Mutations in POLE and survival of colorectal cancer patients--link to disease stage and treatment. *Cancer Med* 2014; 3*:* 1527-1538.

Talhouk A, McConechy MK, Leung S, Li-Chang HH, Kwon JS, Melnyk N*, et al.* A clinically applicable molecular-based classification for endometrial cancers. *Br J Cancer* 2015; 113*:* 299-310.

van de Wetering M, Francies HE, Francis JM, Bounova G, Iorio F, Pronk A*, et al.* Prospective derivation of a living organoid biobank of colorectal cancer patients. *Cell* 2015; 161*:* 933-945.

Wong A, Kuick CH, Wong WL, Tham JM, Mansor S, Loh E*, et al.* Mutation spectrum of *POLE* and *POLD1* mutations in South East Asian women presenting with grade 3 endometrioid endometrial carcinomas. *Gynecol Oncol* 2016; 141*:* 113-120.

Yoshida R, Miyashita K, Inoue M, Shimamoto A, Yan Z, Egashira A*, et al.* Concurrent genetic alterations in DNA polymerase proofreading and mismatch repair in human colorectal cancer. *Eur J Hum Genet* 2011; 19*:* 320-325.

Zehir A, Benayed R, Shah RH, Syed A, Middha S, Kim HR, et al. Mutational landscape of metastatic cancer revealed from prospective clinical sequencing of 10,000 patients. *Nat Med* 2017; 23: 703-713.

Zhao S, Choi M, Overton JD, Bellone S, Roque DM, Cocco E*, et al.* Landscape of somatic single-nucleotide and copy-number mutations in uterine serous carcinoma. *Proc Natl Acad Sci U S A* 2013; 110*:* 2916-2921.

Table S5 P values (Fisher's exact test) for comparison of frequencies of individual *POLE* variants

POLE Variant	D275V	P286H	L424V	P436R	F367S	S459F
P286H	1					
L424V	0.62	0.62				
P436R	0.12	0.12	0.51			
F367S	**0.022**	**0.022**	0.13	0.53		
S459F	**<0.0001**	**<0.0001**	**0.0003**	**0.0069**	0.14	
P286R	**<0.0001**	**<0.0001**	**<0.0001**	**<0.0001**	**<0.0001**	**<0.0001**

Data used for analysis are in Supplementary Table S4. P values indicating statistically significant differences are in bold.

Table S6 Spontaneous mutation rates in diploid yeast strains with *pol2* mutations mimicking human *POLE* variants

Human POLE variant	Yeast *POL2* alleles	Can^R mutation		His^+ reversion		Lys^+ reversion	
		Mutation rate ($x10^{-8}$)[a]	Fold increase	Mutation rate ($x10^{-8}$)[a]	Fold increase	Mutation rate ($x10^{-8}$)[a]	Fold increase
WT	*POL2*/*POL2*	20 (15-24)	1.0	1.1 (0.70-1.7)	1.0	22 (17-26)	1.0
D275V	*pol2-D290V/POL2*	21 (19-23)	1.0	**2.7 (2.0-3.1)**	**2.5**	16 (13-18)	0.73
	pol2-D290V/pol2-D290V	**40 (37-43)**	**2.0**	**5.3 (4.4-5.9)**	**4.8**	22 (19-28)	1.0
P286H	*pol2-P301H/POL2*	**130 (110-150)**	**6.5**	**10 (9.4-13)**	**9.1**	30[b] (27-38)	0.68[b]
	pol2-P301H/pol2-P301H	**270 (240-280)**	**14**	**24 (21-27)**	**22**	49[b] (40-52)	1.1[b]
F367S	*pol2-F382S/POL2*	**230 (190-310)**	**12**	**24 (17-37)**	**22**	59[b] (49-76)	1.3[b]
	pol2-F382S/pol2-F382S	**530 (420-710)**	**27**	**50 (32-69)**	**45**	**66 (37-83)**	**3.0**
L424V	*pol2-L439V/POL2*	**68 (53-82)**	**3.4**	**5.1 (4.5-6.4)**	**4.6**	17 (16-22)	0.77
	pol2-L439V/pol2-L439V	**120 (100-140)**	**6.0**	**13 (11-15)**	**12**	20 (18-22)	0.91
P436R	*pol2-P451R/POL2*	**82 (74-97)**	**4.1**	**7.0 (6.5-9.6)**	**6.4**	20 (17-25)	0.91
	pol2-P451R/pol2-P451R	**180 (150-200)**	**9.0**	**16 (14-19)**	**15**	24 (21-31)	1.1
S459F	*pol2-S474F/POL2*	**500 (370-640)**	**25**	**27 (24-37)**	**25**	**30 (26-31)**	**1.4**
	pol2-S474F/pol2-S474F	**1000 (850-1300)**	**50**	**92 (70-120)**	**84**	**93 (80-120)**	**4.2**

[a]Mutation rates are given as the median of at least 18 independent cultures, with 95% confidence intervals in parentheses. Fold increase is shown relative to the wild-type diploid (WT). Bold text indicates $p < 0.05$ by Wilcoxon-Mann-Whitney compared to WT. Data from this table were used to generate Figure 5.
[b]Strain contains two copies of the *lys2-InsE$_{A14}$* allele (Supplementary Table 1). The fold increase shown is the fold increase in the Lys^+ reversion rate over wild-type divided by two.

Table S7 Spontaneous mutation rates in yeast strains with *pol2-R252H, -R778W* and *-A979V* mutations in MMR-proficient or MMR-deficient (*mlh1*Δ) background

Ploidy	*POL2* allele	*MLH1* allele	Can^R mutation		His^+ reversion		Lys^+ reversion	
			Mutation rate ($x10^{-8}$)[a]	Fold increase	Mutation rate ($x10^{-8}$)[a]	Fold increase	Mutation rate ($x10^{-8}$)[a]	Fold increase
n	*POL2*	*MLH1*	15 (14-20)	1.0	0.92 (0.64-1.3)	1.0	26 (18-30)	1.0
	pol2-R252H	*MLH1*	23 (15-31)	1.5	0.90 (0.69-2.1)	0.98	32 (28-34)	1.2
	pol2-R778W	*MLH1*	15 (13-19)	1.0	0.59 (0.48-0.73)	0.64	14 (14-15)	0.54
	pol2-A979V	*MLH1*	17 (13-34)	1.1	0.93 (0.65-1.2)	1.0	21 (18-24)	0.81
	POL2	*mlh1*Δ	440 (380-490)	29	97 (57-110)	110	130000 (120000-170000)	5000
	pol2-R252H	*mlh1*Δ	**970 (930-1100)**	**65**	**150 (140-160)**	**160**	160000 (140000-260000)	6200
	pol2-R778W	*mlh1*Δ	530 (440-650)	35	100 (91-110)	110	140000 (130000-160000)	5400
	pol2-A979V	*mlh1*Δ	410 (390-470)	27	99 (90-110)	110	170000 (140000-190000)	6500
2n	*POL2*/*POL2*	*MLH1*/*MLH1*	17 (16-23)	1.0	1.2 (0.87-1.6)	1.0	18 (16-22)	1.0
	pol2-R252H/POL2	*MLH1*/*MLH1*	18 (15-21)	1.1	1.2 (1.0-1.7)	1.0	20 (17-24)	1.1
	POL2/*POL2*	*mlh1*Δ/*mlh1*Δ	450 (410-520)	26	110 (98-140)	92	120000 (100000-130000)	6700
	pol2-R252H/POL2	*mlh1*Δ/*mlh1*Δ	**820 (710-1200)**	**48**	**150 (140-170)**	**130**	140000 (120000-160000)	7800

[a]Mutation rates are given as the median of at least 18 independent cultures, with 95% confidence intervals in parentheses. Fold increase is shown relative to the wild-type haploid or diploid. Bold text indicates $p < 0.05$ by Wilcoxon-Mann-Whitney compared to *mlh1*Δ haploids or *mlh1*Δ/*mlh1*Δ diploids. Data from this table were used to generate Figure 6.

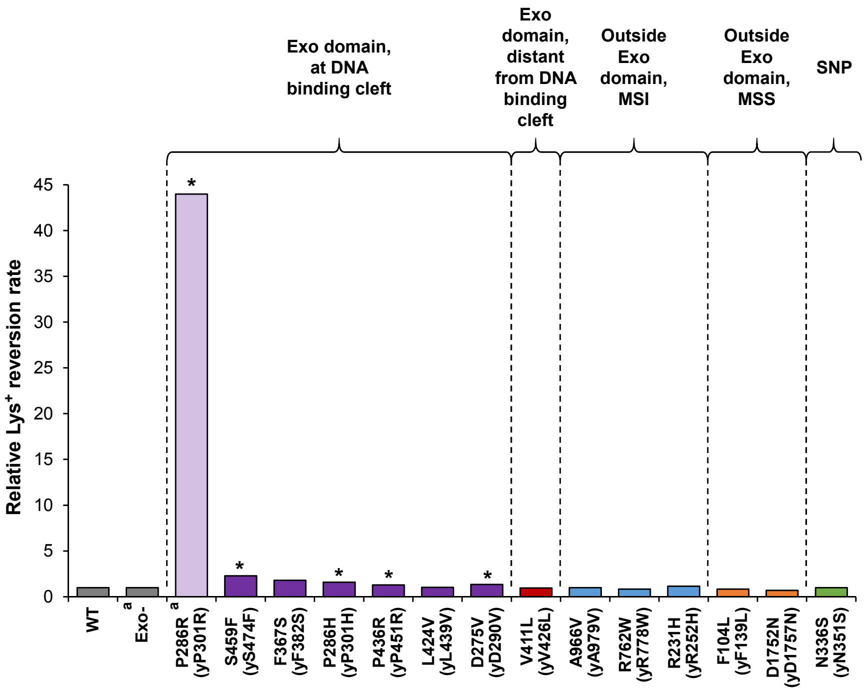

Figure S1 Effect of cancer-associated Polε variants on the rate of *lys2-InsE*$_{A14}$ reversion in haploid yeast strains. Relative mutation rates are shown compared to wild-type (WT). All experimental details and symbols are as in Figure 2. Asterisks indicate $p < 0.05$ by Wilcoxon-Mann-Whitney compared to WT. Data are from Supplementary Table S3. [a]Data from Kane and Shcherbakova 2014.

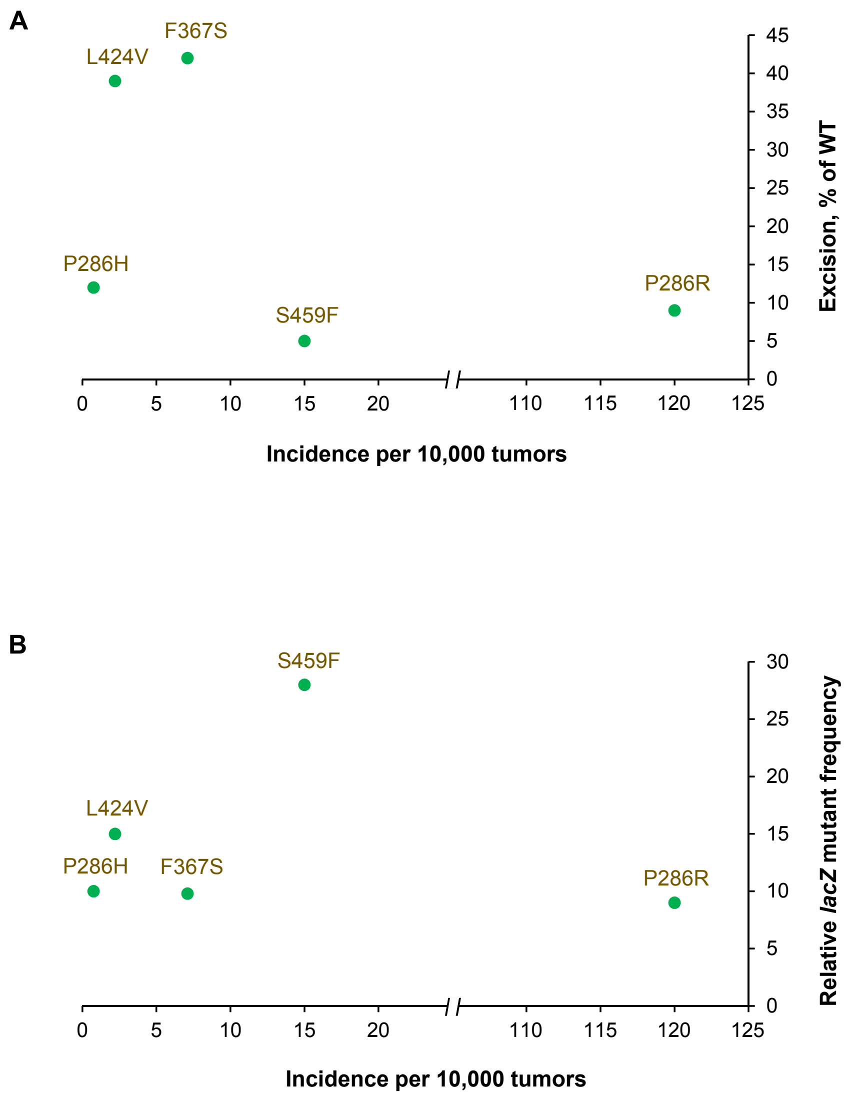

Figure S2 Lack of correlation between *in vitro* effects of Polε mutations and their frequency in tumors. [A] Relationship between exonuclease activity defect and variant frequency. [B] Relationship between *in vitro* error rate and variant frequency. Exonuclease activity and *in vitro* error rates were measured in Shinbrot *et al.* 2014 using the N-terminal fragment of human Polε containing the exonuclease and DNA polymerase domains. The variant frequency in tumors was determined as described in Figure 4.

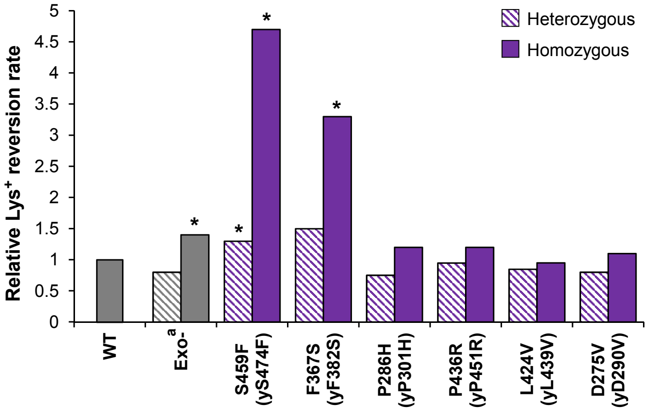

Figure S3 Effect of cancer-associated Polε variants on the rate of *lys2-InsE$_{A14}$* reversion in diploid yeast strains. The strains contained one copy of the *lys2-InsE$_{A14}$* reporter allele and a nonrevertible mutation at the same position of the *LYS2* gene in the homologous chromosome. All other experimental details and symbols are as in Figure 5. Relative mutation rates are shown compared to wild-type (WT). Asterisks indicate $p < 0.05$ by Wilcoxon-Mann-Whitney compared to WT. Data are from Supplementary Table S6. [a]Data from Kane and Shcherbakova 2014.

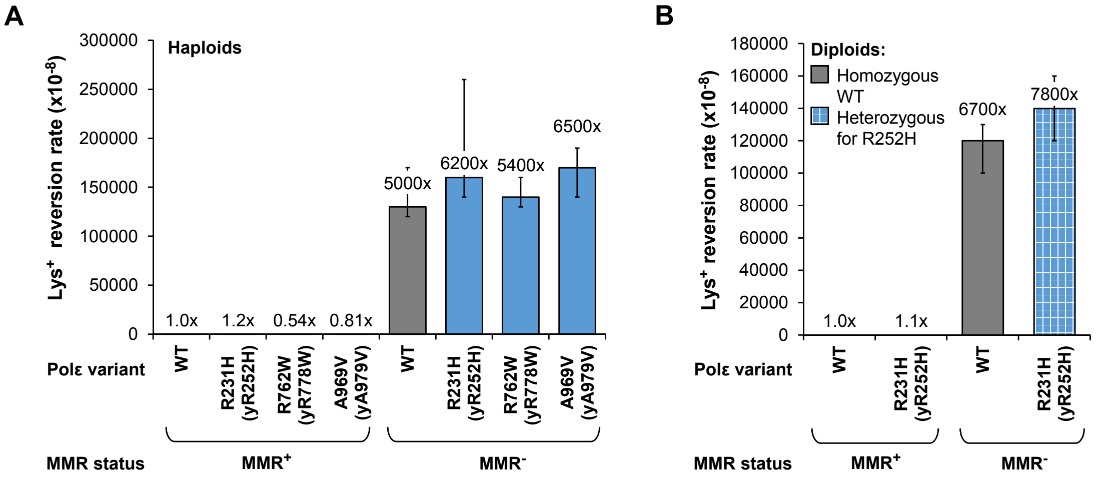

Figure S4 Polε variants found in MMR-deficient tumors do not affect the rate of *lys2-InsE*$_{A14}$ reversion in MMR-proficient or MMR-deficient background. The reversion rate was measured in haploid [A] or diploid [B] yeast strains containing indicated chromosomal *pol2* mutations. The MMR defect was mimicked by deleting the yeast *MLH1* gene. Mutation rates are given as the median for at least 18 independent cultures, with error bars indicating 95% confidence intervals. Fold increase in mutation rate relative to the strain with wild-type *POL2* and *MLH1* genes is shown above each bar. Data are from Supplementary Table S7.
